# A UK wide survey of general surgeons' experience of the primary repair of obstetric anal sphincter injuries

**DOI:** 10.1111/codi.17244

**Published:** 2024-11-20

**Authors:** Nada Elsaid, Gregory P. Thomas, Emma V. Carrington, Ruwan J. Fernando, Carolynne J. Vaizey

**Affiliations:** ^1^ St Mark's the National Bowel Hospital London UK; ^2^ Imperial College London London UK; ^3^ Imperial College Healthcare NHS Trust London UK

**Keywords:** anal sphincter, faecal incontinence, obstetric anal sphincter injury, primary sphincter repair

## Abstract

**Aim:**

Obstetric anal sphincter injuries (OASIs) are associated with devastating consequences, mainly faecal incontinence. A timely and correct repair is necessary to reduce the risk of maternal morbidity. The aim was to explore the experience and practice of on‐call general surgeons in the acute repair of OASIs.

**Method:**

A cross‐sectional, observational questionnaire study was performed. Registrars and consultants participating in an emergency general surgical rota in the UK were included. A 33‐item questionnaire was disseminated over a 9‐month period from April 2023. A descriptive, thematic analysis of the data was undertaken.

**Results:**

In all, 310 responses were analysed. 42.3% of colorectal respondents (of which 29% were pelvic floor specialists), 24.3% of general surgeons, 16.7% of hepato‐biliary surgeons and 13.7% of upper gastrointestinal surgeons were contacted to assist with an acute repair. Of those contacted, 52.3% typically assisted with a 3C or 4 tear, 54.2% received no training and 95.5% performed less than three acute repairs in the previous year. 57.6% of all respondents were not confident at all in the repair of these injuries, 55% highlighted a lack of experience and 36% mentioned a curricular gap.

**Conclusion:**

Surgeons may be called to assist with an acute OASI repair, particularly in cases of severe anatomical disruption. This occurs infrequently. There is a lack of consensus as to who is responsible for these injuries. Obstetricians have structured training in both the recognition and repair of these injuries. This paper serves to highlight the lack of training for surgeons who report doing this surgery despite lacking the required competences.

**What does this paper add to the literature?**

The paper highlights the experience, knowledge and training of emergency general surgeons in the acute repair of an obstetric anal sphincter injury, a potentially devastating injury.

## INTRODUCTION

Perineal tears occur in 80%–90% of vaginal deliveries [1, 2]. Third‐ and fourth‐degree tears involve the anorectal sphincter complex and are known as obstetric anal sphincter injuries (OASIs) [3]. OASIs may be associated with a myriad of complications including incontinence, rectovaginal fistulas, pelvic pain and prolapse [4, 5]. The highest incidence of OASIs (19.4%) has been reported in primiparous women who have undergone a forceps delivery [6].

To improve maternal outcomes, prompt recognition and repair of these injuries is advocated post‐partum, with the consensus being that repair should be undertaken as soon as possible and within 12 h of delivery [7, 8, 9]. Satisfactory repair is crucial in minimising the risk of maternal morbidity [10]. The persistence of sphincter defects following primary repair is associated with greater incontinence scores as well as a greater risk of long‐term complications [11, 12, 13]. Furthermore, although an attempt at secondary sphincteroplasty may lead to improved continence in the short term, the positive effects of secondary repair appear to wane with time [14, 15].

Although perineal injury is a recognised complication of vaginal delivery, a failure to adequately repair the injury may also carry medicolegal implications as well as increase the financial and socioeconomic burden relating to the management of the sequelas of these injuries [16, 17, 18].

To date, there is little consensus on who should perform the primary repair of OASIs. The Royal College of Obstetricians and Gynaecologists (RCOG) state that the repair should be undertaken by ‘appropriately trained practitioners’ and that ‘involvement of a colorectal surgeon will be dependent on local protocols, expertise and availability’ [19]. Experience in repair has been shown to be an important factor in determining the success of a primary repair [20].

A previous national survey of practice amongst UK obstetricians and coloproctologists identified a wide variation in experience, methods of repair, follow‐up and recommendations for future delivery. In this survey, only 40% of coloproctologists were involved in the acute repair of these injuries, in comparison to almost 90% of obstetricians [21].

As these injuries could happen at any time of day and in the absence of a dedicated colorectal OASI on‐call rota, the authors queried whether the on‐call emergency surgeon may be contacted instead to facilitate a timely repair of these injuries. The aim of this study was to explore an on‐call general surgeons' experience, knowledge and current practice in relation to the acute repair of OASIs.

## METHODS

A cross‐sectional, observational study was performed. Ethics approval was not required as per the Health Research Authority. Confirmation of capacity and capability was successfully attained at London Northwest University Healthcare Trust prior to study commencement. The study was registered on ClinicalTrials.gov, Protocol Registration and Results System (NCT05898945).

An anonymous online, open survey was constructed using the Qualtrics survey tool, which was accessed via Imperial College University. The inclusion criteria for participation were registrars or consultants, of any specialist background, who cover a general surgical on‐call rota and who were currently practising in the UK. Participants provided implied consent by voluntarily completing the survey. They were not provided any remuneration for this study. The exclusion criteria included starting a survey but failing to submit it and not fulfilling the inclusion criteria.

The questions were based on best practice guidance as outlined by the RCOG Green‐top Guideline No. 29 on the management of third‐ and fourth‐degree perineal tears [19]. A clinician involvement group, comprising emergency and colorectal surgeons as well as obstetricians, was consulted regarding the study design, to ensure that the study reflected relevant and clear content that fulfilled the aims and objectives.

Adaptive questioning was used. Responders were only shown relevant questions, based on their responses to earlier questions. The survey was presented as seven slides, with a minimum of 25 and a maximum of 33 items. The question order was not randomised. To minimise the risk of ‘guessing’ an answer, respondents were given the options of ‘unsure’ and ‘other (please specify)’. The survey was tested by a group of 20 registrars and consultants and amended accordingly prior to widespread dissemination. The average time for completion of the survey was less than 5 min. A convenience sample was used.

The survey was disseminated to members of the Association of Surgeons of Great Britain and Ireland (ASGBI) and the Association of Coloproctology of Great Britain and Ireland (ACPGBI). It was also sent via social media platforms, including Twitter and LinkedIn, and distributed via the higher general surgical registrar trainee groups on social software chat groups (Telegram and WhatsApp). To further optimise recruitment, participants were encouraged to forward the survey onto their colleagues and the clinical directors of individual hospitals were also emailed separately. Distribution began in April 2023. Reminders were sent out periodically over a 9‐month period, after which the survey was closed in January 2024.

The Checklist for Reporting Results of Internet E‐Surveys (CHERRIES) tool was adhered to, to ensure transparency of the methodology. It was not possible to calculate the total number of surgeons that the survey was sent to and therefore the response rate as it was circulated using a myriad of social platforms and a chain‐referral sampling method was used. A descriptive analysis of the quantitative data was made on the Qualtrics platform. A thematic analysis of the qualitative data was also performed. The questionnaire is shown in the Appendix.

## RESULTS

In total, there were 324 submitted responses. Ten responses were excluded as they were incomplete. Four responses were excluded from the dataset as the responders were practising abroad (Italy, Saudi Arabia, New Zealand and the Middle East) at the time of completion of the survey. A total of 310 responses were included in the final data analysis.

### Surgeon demographics

This is shown in Table 1. Almost 80% of respondents were under the age of 50. Over half (52.9%) of all respondents were consultants. 65.8% of the consultants had been in post for over 5 years. 70.7% of the trainees were ST5 or above. 60.4% of all participants had attained the Fellowship of the Royal College of Surgeons (FRCS) exit examination. Over half (55.6%) were colorectal specialists and 15.4% had a specialist interest in pelvic floor surgery. 42.3% were practising in London at the time of completion of the survey.

**TABLE 1:** Surgeon demographics.

Demographics	Number (%)
Age	
<40	138 (44.5)
40–49	109 (35.2)
50–59	51 (16.5)
60+	12 (3.9)
Did not answer	0
Grade/designation	
Trainee^a^	115 (37.1)
ST3/ST4	29 (29.3)
ST5/ST6	27 (27.3)
ST7/ST8/Peri or Post CCT	43 (43.4)
Did not answer	16
SAS^b^	31 (10)
Consultant	164 (52.9)
0–4 years	51 (34.2)
5–9 years	48 (32.2)
10–19 years	32 (21.5)
20–29 years	17 (11.4)
≥30 years	1 (0.7)
Did not answer	15
Did not answer	0
FRCS^c^ exit examination	
Yes	177 (60.4)
No	116 (39.6)
Did not answer	17
Sub‐speciality	
General	37 (12.6)
Upper gastrointestinal	51 (17.4)
Colorectal	163 (55.6)
Hepato‐biliary	18 (6.1)
Other (breast, oncoplastic, transplant, vascular, trauma, urology, endocrine)	24 (8.2)
Did not answer	17
Specialist in pelvic floor surgery^d^	
Yes	45 (15.4)
No	247 (84.6)
Did not respond	18
Region currently practising in	
East of England	32 (11.0)
London	123 (42.3)
Midlands	27 (9.3)
North East and Yorkshire	18 (6.2)
North West	23 (7.9)
South East	29 (10.0)
South West	11 (3.8)
Scotland	12 (4.1)
Wales	7 (2.4)
Northern Ireland	4 (1.4)
Crown Dependencies	1 (0.3)
United Kingdom (unknown location)	4 (1.4)
Did not respond	19

^a^ The UK higher surgical training programme comprises a 6‐year programme (ST3–ST8), culminating in the certificate of completion of training (CCT) and registration on the General Medical Council specialist register.

^b^ SAS, Speciality and associate specialist doctor.

^c^ FRCS, Fellowship of the Royal College of Surgeons, a knowledge and competence‐based examination that is completed at the end of speciality training.

^d^ Participants were allowed to decide this at their own discretion.

### Surgeon experience in the primary repair of OASIs

Almost a third (31.5%) of all respondents had been contacted by an obstetrician during an on‐call to assist with the primary repair of an OASI. Table 2 delineates the characteristics and experience of this group. Almost half (42.3%) of all colorectal respondents had been contacted at some point in their careers to assist with an acute repair. Only 29.0% of this group were pelvic floor specialists. 24.3% of general surgeons, 16.7% of hepato‐biliary surgeons and 13.7% of upper gastrointestinal surgeons were contacted by an obstetrician. 16% of trainee respondents (all of whom were ST5 or above) had been involved in primary repairs, compared to 43.3% of consultants. 52.3% were contacted to assist with a 3C/4 tear. 95.5% had performed fewer than three acute repairs in the previous year. Over half (54.2%) of those contacted had not received any training in the repair of an acute OASI.

**TABLE 2:** Characteristics and experience of surgeons assisting with primary repair of OASIs.

Sub‐speciality	Number (%)
General	9 (24.3)
Upper gastrointestinal	7 (13.7)
Colorectal	69 (42.3)
Pelvic floor specialist	20 (29.0)
Non‐pelvic floor specialist	49 (71.0)
Hepato‐biliary	3 (16.7)
Other (breast, breast oncoplastic)	3 (12.5)
Did not respond	1
Pelvic floor specialist	
Yes	20 (44.4)
Grade	
Trainee	19 (16.5)
SAS	2 (6.5)
Consultant	71 (43.3)
Grade of tear typically asked to repair	
3A/B	10 (11.4)
3C	6 (6.8)
4	40 (45.5)
All grades	32 (36.4)
Did not respond	4
Number of repairs performed in previous year	
0	54 (61.4)
1–2	30 (34.1)
3–4	1 (1.1)
5–10	1 (1.1)
>10	2 (2.3)
Did not respond	4
Training received	
Yes	38 (45.8)
No	45 (54.2)
Did not respond	9

Abbreviation: SAS, Speciality and associate specialist doctor.

**FIGURE 1:**
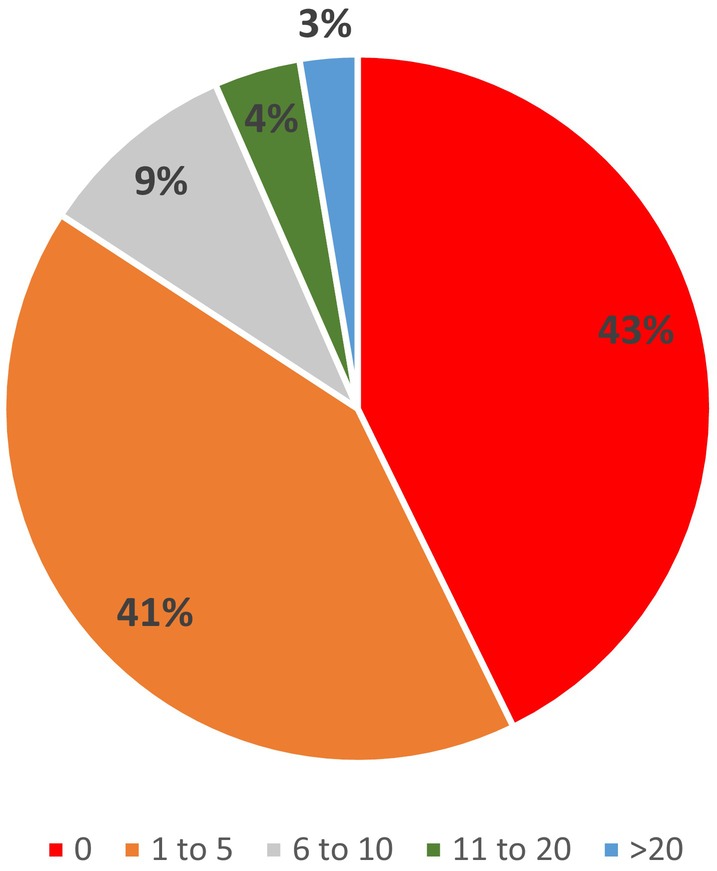
Total number of primary OASI repairs undertaken by consultants.

Figure 1 demonstrates the total number of primary repairs performed by consultants throughout their careers. The results show that 84% of consultants had performed 0–5 repairs. Almost half of the colorectal respondents (49.4%) had not performed any in their careers.

### Confidence, training and knowledge in primary repair

In all, 43.2% of those who had completed the FRCS exit examination were not confident at all in acute repair whilst only 6.8% were completely confident. The latter group comprised solely colorectal specialists, of which 70% were pelvic floor specialists. 57.6% of all the respondents were not confident at all in the repair of these injuries. 78.3% of all respondents had not received any form of training whilst 21.7% reported that they had undergone training for the repair of these injuries.

**FIGURE 2:**
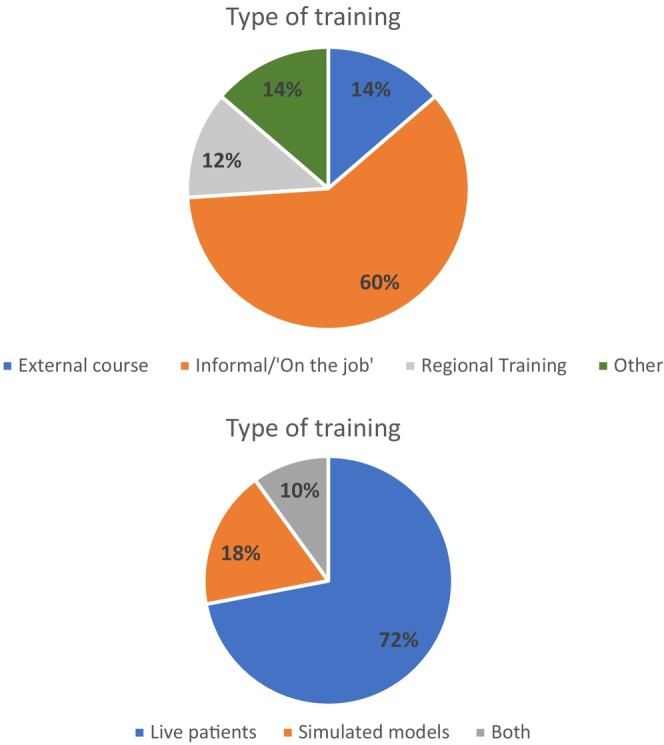
Types of training undertaken by surgeons.

Figure 2 illustrates the type of training undertaken, where ‘other’ refers to training received in advanced colorectal fellowships whilst abroad and as a trainee in obstetrics and gynaecology (prior to career change to general surgery). The respondents were allowed to ‘tick all that apply’. The findings show that most of the training comes from informal or ‘on the job’ experience and on live patients.

When asked regarding the timing of primary repair, almost half (48%) of the respondents advocated an immediate repair post‐partum, 13.8% believed that a repair would be undertaken whenever a colorectal specialist was available and 34.5% were unsure.

Interestingly, the majority (43.2%) believed that a full‐thickness external anal sphincter (EAS) tear should be repaired using the overlapping method only with only 8.8% advocating an end‐to‐end repair and 18.9% proclaiming that either technique could be used with no deleterious consequences (Table 3). 33.1% believed that a partial‐thickness EAS tear should be repaired using the end‐to‐end technique. 33.8% would consider an overlapping repair of a partial‐thickness EAS tear. Almost a third (32.4%) would use figure of eight sutures to stem bleeding in the rectal mucosa (Table 3).

**TABLE 3:** Knowledge of surgeons who have attained the FRCS.

How to repair a full‐thickness EAS tear	
Overlapping	64 (43.2)
End‐to‐end	13 (8.8)
Overlapping or end‐to‐end with no difference in outcome	28 (18.9)
Unsure	43 (29.1)
Did not respond	29
How to repair a partial‐thickness EAS tear (all 3A and some 3B tears)	
Overlapping	26 (17.6)
End‐to‐end	49 (33.1)
Overlapping or end‐to‐end with no difference in outcome	24 (16.2)
Unsure	49 (33.1)
Did not respond	29
In relation to EAS, how to repair a torn IAS if identified	
Together	11 (7.5)
Separately	89 (60.5)
Unsure	47 (32.0)
Did not respond	30
Suture material used for repair of IAS/EAS	
Vicryl Rapide	2 (1.4)
Vicryl or PDS	100 (67.6)
Non‐absorbable	6 (4.1)
Unsure	40 (27.0)
Did not respond	29
Use of figure of eight sutures to stem bleeding in rectal mucosa	
Yes	48 (32.4)
No	46 (31.1)
Unsure	54 (36.5)
Did not respond	29

Abbreviations: EAS, external anal sphincter; FRCS, Fellowship of the Royal College of Surgeons; IAS, internal anal sphincter; PDS, polydioxanone.

**FIGURE 3:**
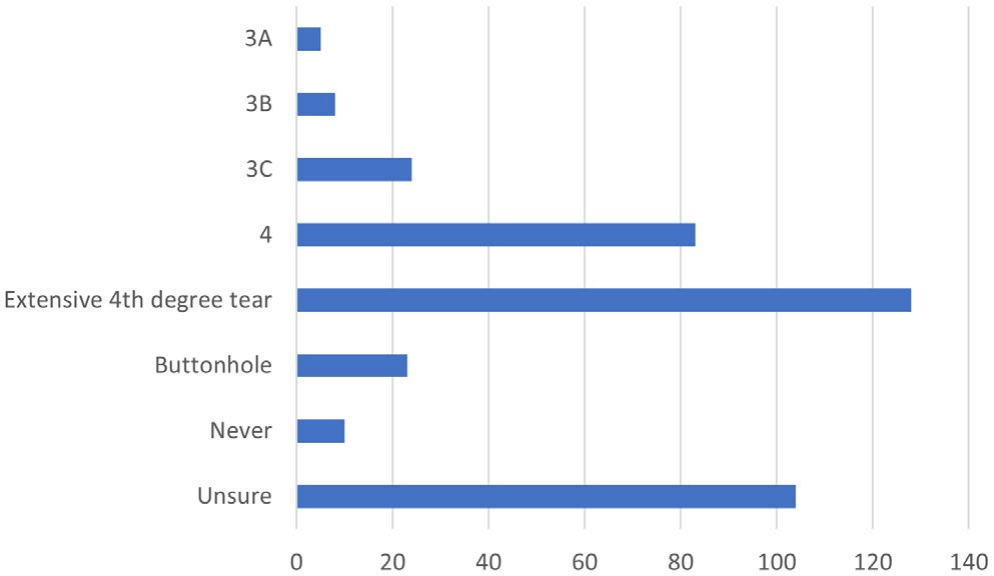
When to defunction with a stoma.

Figure 3 shows the responses of surgeons when asked about the circumstances under which they would consider a stoma. The results show that, the greater the grade of the injury, the more likely that a stoma was considered. 34.5% believed that a stoma would be indicated with a Grade 3C or 4 tear, 7.4% believed that a stoma would be required in a buttonhole injury and a third were unsure.

**FIGURE 4:**
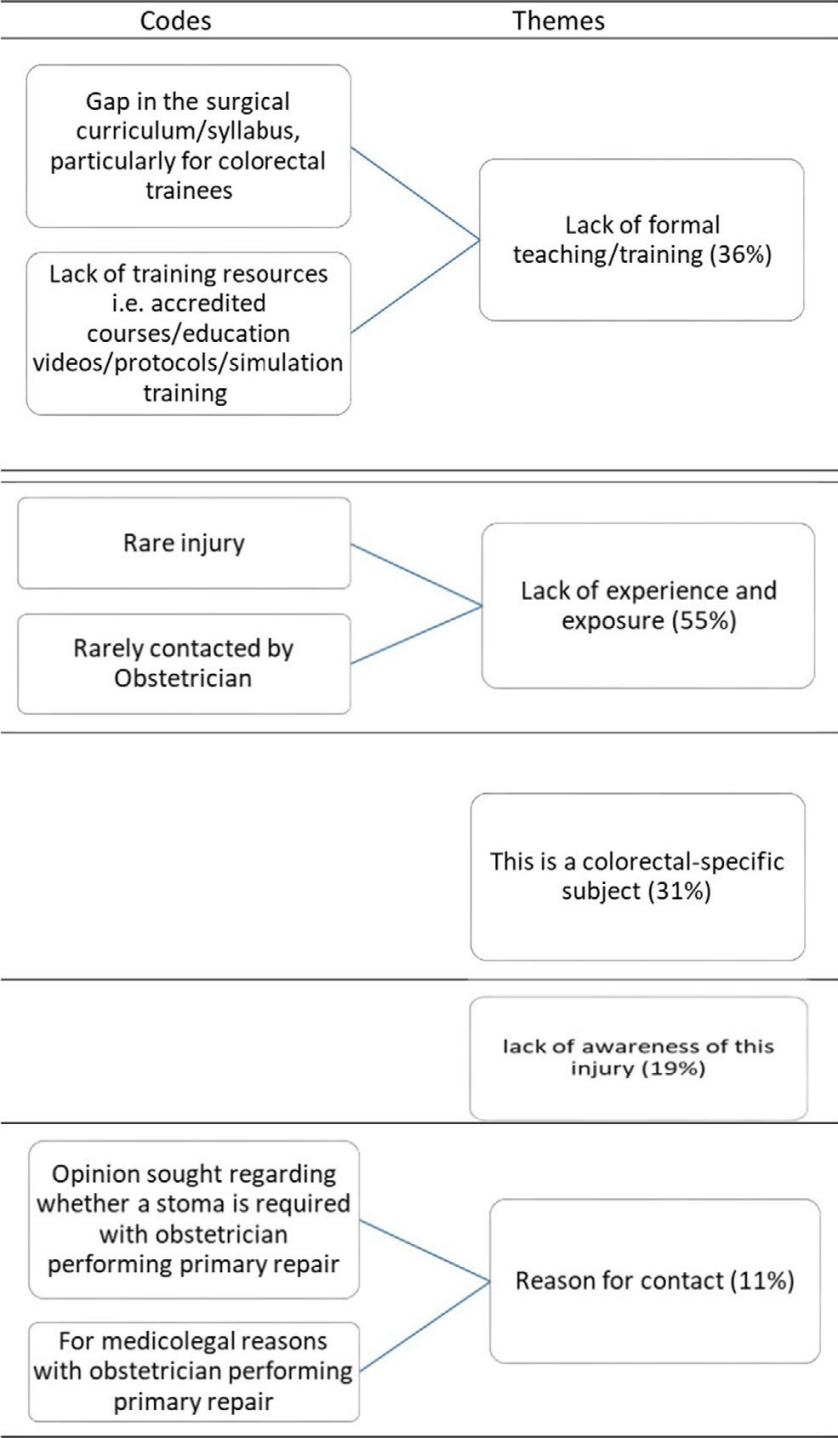
Surgeon comments on the primary repair of OASIs.

Figure 4 refers to the themes that emerged when participants were asked to make any additional comments in relation to the primary repair of OASIs by surgeons.

55% had highlighted the lack of experience and exposure that surgeons have in the acute repair of OASI. This was thought to be due to the infrequent contact with these injuries. 36% of responders highlighted the gap in the surgical curriculum and lack of formal training and educational opportunities and resources. 11% had highlighted the reason for contact as being for advice regarding whether a stoma was indicated or for medicolegal reasons.

## DISCUSSION

Despite the intensive training of obstetricians in the recognition and repair of OASIs in the UK, surgeons covering an emergency rota may be asked to assist in the acute repair of an OASI. These results show that this is especially the case for the more severe cases of anatomical disruption: the Grade 4 tears. This occurs infrequently, a finding that is supported by the study by Oh et al., which showed that only 8.6% of colorectal surgeons were called routinely to perform an acute repair of a fourth‐degree tear [22].

The results demonstrate that emergency surgeons of any specialist background may be contacted, perhaps because an acute OASI may happen at any time of the day and a timely repair is advocated. 48% of respondents advocated an immediate repair post‐partum. The majority (55%) of colorectal consultants in the Roper et al. paper had also advocated for this [9]. The obstetrician may therefore resort to contacting the emergency on‐call surgeon due to accessibility and for general surgical advice. Nevertheless, the results did show that colorectal specialists are contacted more commonly compared to other sub‐specialities, probably due to their involvement in the elective repair of sphincters and their sub‐speciality expertise in the anatomy of the rectum and anal canal.

Whilst a delay to repair for a day or so to await the availability of a pelvic floor surgeon or even transfer to another hospital is feasible and considered safe in terms of physical outcome, this may not be the optimal pathway for a new mother [23, 24, 25].

The findings also demonstrated that surgical consultants were more likely to be contacted than trainees or SAS grade doctors. This may be secondary to surgeons being asked to assist most often with the more complex cases of OASIs or because of the morbidity associated with sub‐standard repair and associated litigation risk meaning that the most senior physician is typically contacted. In contrast, Ismail demonstrated that the majority of obstetricians performing a primary repair were middle grade (76%), with only 2.9% performing a repair at consultant level [26].

The findings had also shown that a surgeon may be asked to be present during the repair of an OASI for ‘medicolegal’ reasons. Interestingly, a referral to colorectal surgeons has been shown to protect obstetricians in litigations of malpractice relating to OASIs [27].

Regarding repair of a full‐thickness EAS tear, similar findings were reported by Roper et al. [9] with 70% of colorectal consultants preferring an overlapping repair, 17.5% end‐to‐end and 12.5% using either method. The overlapping method had been introduced in a bid to reduce the sphincter defects that were identified via endoanal ultrasound following end‐to‐end repair of the sphincter [28, 29]. However, evidence to date is insufficient to conclude that one technique is superior to the other and guidelines state that either method could be used at the clinician's discretion [8, 30].

Almost a third would consider an overlapping repair for a partial‐thickness EAS tear. It is not possible to overlap a partially damaged muscle. Excessive mobilisation, dissection and disruption of surrounding anatomy would be necessary to free the ends to facilitate a tension‐free overlapping repair which is not generally recommended [19, 29]. Over half of those who were post FRCS would use either Vicryl or PDS in repairing the anal sphincter. A randomised controlled trial has shown no significant difference in use of Vicryl or PDS in internal anal sphincter (IAS)/EAS repair [31]. 60.5% believed that the IAS should be repaired separately to the EAS. Adequate repair of the IAS is fundamental in reducing the risk of incontinence [13]. A third of those who had attained the FRCS would use figure of eight sutures to stem bleeding from the rectal mucosa. This technique is not recommended as it may predispose to tissue ischaemia and possible rectovaginal fistula formation [19].

Although there is no evidence that routine use of a stoma confers any benefit in the immediate period after sustaining an OASI and prior to acute repair, this study demonstrated a wide variation in opinion among surgeons as to when a stoma is indicated. It is the authors' opinion that a covering stoma is very rarely needed following repair of these injuries.

When asked about the existence of a policy for the management of OASIs at their hospital trust, 87.3% were either unsure or reported that there was no such policy in place. Only 12.7% were aware of the existence of a guideline. The RCOG guidance states that ‘units should have a clear protocol for the management of OASIs’ and evidence has shown that the existence of a policy enhances the outcomes of these injuries [19, 32].

A strength of this study is that it is the largest UK National Survey exploring the experience of surgeons in the acute repair of this potentially devastating injury. The findings also reflect current clinical practice as those who may have had historical experience in OASIs were excluded from the study. It is also the first study that evaluates non‐colorectal surgeons’ as well as trainees’ experience in primary repair of obstetric sphincter injuries. It is the first study that demonstrates that non‐colorectal surgeons may be contacted for assistance with primary repair of OASI.

A possible limitation of this study is that it did not ask about the experience and exposure of surgeons in secondary repair of OASIs and in repair of sphincter injury due to non‐obstetric causes (e.g., secondary to sexual intercourse, pelvic trauma and iatrogenic injury). However, it could be argued that the surgical conditions in the different circumstances differ, thereby requiring a separate proficiency in both.

Despite the mass dissemination of the survey within the general surgical community, the authors note that more than half of the responders (55.6%) were of colorectal background. This may be secondary to a bias relating to web‐based surveys, the so‐called volunteer effect which describes the self‐selection of participants. Colorectal specialists may be more inclined to complete a survey relating to a colorectal topic than those who do not have expertise or interest in the subject. The survey was also distributed via ACPGBI which is a society of coloproctologists. However, other non‐colorectal platforms were also utilised for the distribution of the survey as outlined in the Methods section.

As a response rate could not be calculated, the results observed may not be generalisable to the population studied [33]. Data were collected over a period of 9 months to allow time for the authors to distribute the survey via the different platforms to the wider surgical community and to allow sufficient time for the respondents to complete the survey, with the potential to enhance the response rate.

The consensus across all ages and sub‐specialities was that colorectal surgeons should be involved in the primary repair of OASIs, with only 18.5% stating that obstetricians should repair these injuries alone. Interestingly, this was also the consensus among the group with no prior training in primary repair. These findings differed to the literature. In a study by Roper et al., 55% of colorectal surgeons stated that obstetricians should perform a primary repair without any colorectal input [9]. The study by Fernando et al. found that only 19% of coloproctologists stated that they should be involved in primary repair [21]. Several authors have suggested that the follow‐up of these patients should be led by the colorectal specialists, irrespective of who performed the primary repair [26, 34, 35].

The consensus (81.9%) was that all OASI patients should be followed up in a dedicated perineal clinic with endoanal ultrasound and anal manometry. This is particularly important when planning the mode of future deliveries as abnormal anorectal investigations, even in the absence of symptoms, may favour a recommendation for an elective caesarean section [19].

Interestingly, almost a third of pelvic floor specialists (32.5%) were unaware of the RCOG Green‐top Guideline relating to the management of third‐ and fourth‐degree perineal tears, perhaps highlighting the need for a greater collaboration between the obstetricians and coloproctologists to enhance patient outcomes. A multi‐disciplinary approach towards training and the management of these patients has been previously advocated [9, 35].

While 80% of respondents in this study had not received any form of training, a similar survey of obstetricians revealed that only 20.5% of obstetricians had received no training [36]. In the paper by Roper et al., the majority of colorectal consultants (70%) believed that colorectal surgeons should receive training in the acute repair of OASI [9]. In a survey of colorectal surgeons in Australia and New Zealand, 60.6% reported that they had either never been exposed to or rarely been exposed to OASIs during their fellowship training [22].

## CONCLUSION

There remains a lack of consensus on who is responsible for the repair of these injuries. On‐call general surgeons may assist with an acute OASI repair, particularly in cases of severe anatomical disruption. Owing to the potential consequences of sub‐standard sphincter repair, surgeon involvement should only occur if adequate exposure and training is assured.

This study shows there is a lack of training, competence and confidence with primary OASI repair among both general and colorectal surgeons. Either obstetricians should repair these injuries or training should be expanded to the surgeons, who may be called on to suture these life changing injuries.

## AUTHOR CONTRIBUTIONS

**Nada Elsaid:** Conceptualization; methodology; data curation; formal analysis; writing – original draft. **Gregory P. Thomas:** Conceptualization; methodology; data curation; writing – review and editing; supervision. **Emma V. Carrington:** Writing – review and editing; supervision; data curation. **Ruwan J. Fernando:** Methodology; writing – review and editing; supervision. **Carolynne J. Vaizey:** Conceptualization; methodology; data curation; writing – review and editing; supervision.

## FUNDING INFORMATION

This research received no specific grant from any funding agency in the public, commercial or not‐for‐profit sectors.

## CONFLICT OF INTEREST STATEMENT

None.

## ETHICS STATEMENT

Health Research Authority approval was sought for this study but found not to be required. Confirmation of capacity and capability was successfully attained at London Northwest University Healthcare Trust prior to study commencement.

## CLINICAL TRIAL REGISTRATION

The study was registered on ClinicalTrials.gov, Protocol Registration and Results System (NCT05898945).

## Data Availability

The data that support the findings of this study are available from the corresponding author upon reasonable request.

## SURVEY

1. What is your age group?
  ☐ <40 ☐ 40–44 ☐ 45–49 ☐ 50–54 ☐ 55–59 ☐ 60+
2. What is your designation?
  ☐ Trainee (please specify grade) ______
  ☐ SAS (please specify) _____
  ☐ Consultant (please specify years of experience as a consultant) _______
3. Have you been awarded the FRCS?
  ☐ Yes ☐ No
4. Which region do you currently practise in?
  ☐ East of England ☐ London ☐ Midlands ☐ North East and Yorkshire ☐ North West ☐ South East
  ☐ South West ☐ Other (please specify) _____
5. Which deanery did you complete your fellowship training at?
  ☐ East Midlands (North) ☐ East Midlands (South) ☐ East of England ☐ Kent, Surrey and Sussex
  ☐ North Central and East London ☐ North West London ☐ South London (South East)
  ☐ South London (South West) ☐ North East ☐ North West (Mersey) ☐ North West ☐ South West (Peninsula) ☐ South West (Severn) ☐ Thames Valley ☐ Wessex ☐ West Midlands ☐ Yorkshire and the Humber ☐ Ireland ☐ Northern Ireland ☐ Wales ☐ Scotland (East – Dundee) ☐ Scotland (North – Aberdeen) ☐ Scotland (South East – Edinburgh) ☐ Scotland (West – Glasgow) ☐ Abroad ☐ Other ________
6. What is your sub‐speciality of expertise?
  ☐ General ☐ Upper GI ☐ Colorectal ☐ Hepato‐biliary ☐ Other (please specify) ____________
7. Do you have a specialist interest in pelvic floor surgery?
  ☐ Yes ☐ No
8. Have you ever been contacted by an obstetrician during your on‐call to assist with the repair of an OASI?
  ☐ Yes ☐ No
9. If you answered yes to question 8, how many primary OASI repairs have you performed over the last year?
  ☐ 0 ☐ 1–2 ☐ 3–4 ☐ 5–10 ☐ Other (please specify)_________
10. What is the total number of primary OASI repairs you have undertaken in your career?
  ☐ 0 ☐ 1–5 ☐ 6–10 ☐ 11–20 ☐ Other(please specify)______
11. What grade of tear are you typically asked to repair?
  ☐ 3A/3B ☐ 3C ☐ 4 ☐ All Grades
12. In your experience, when are repairs typically undertaken?
  ☐ Immediately post‐partum ☐ Next day ☐ Whenever a colorectal specialist is available
13. Are you aware of the RCOG Green‐top Guideline of 2015, titled the management of third‐ and fourth‐degree perineal tears?
  ☐ Yes ☐ No
14. Is there a guideline/protocol/policy that exists in your hospital for the management of obstetric anal sphincter injuries?
  ☐ Yes ☐ No ☐ Unsure
15. Have you received any training to repair such injuries?
  ☐ Yes ☐ No.
16. If you answered yes to question 15, please detail the type of training you have received *(Tick all that apply)*
  ☐ External course ☐ ‘Informal/On the job’ ☐ Regional Training ☐ Abroad ☐ Other (please specify) _________
17. How confident are you in repairing obstetric anal sphincter injuries?
  Least confident ☐ 1 ☐ 2 ☐ 3 ☐ 4 ☐ 5 Most confident
18. Under which circumstances would you consider defunctioning with a stoma? *(Tick all that apply)*
  ☐ 3A ☐ 3B ☐ 3C ☐ 4 ☐ Extensive fourth degree tear ☐ Buttonhole ☐ Never ☐ Unsure
19. How would you repair a full‐thickness EAS tear?
  ☐ Overlapping ☐ End‐to‐end ☐ Overlapping or end‐to‐end with equivalent outcomes ☐ Unsure
20. How would you repair a partial‐thickness EAS tear (all 3A and some 3B tears)?
  ☐ Overlapping ☐ End‐to‐end ☐ Overlapping or end‐to‐end with equivalent outcomes ☐ Unsure
21. If the torn IAS can be identified separately, do you repair both muscles together or separately?
  ☐ Together ☐ Separately ☐ Unsure
22. Would you use figure of eight sutures to stem bleeding in the rectal mucosa?
  ☐ Yes ☐ No ☐ Unsure
23. Which suture material do you use for repair of the IAS/EAS?
  ☐ Vicryl rapide ☐ Vicryl or PDS ☐ Non‐absorbable ☐ Unsure
24. Who, in your opinion, should repair Grade 3 and 4 perineal tears?
  ☐ Colorectal surgeon ☐ Obstetrician ☐ Both together
25. How would you follow up these patients?
  ☐ Dedicated perineal clinic with endoanal ultrasound and anal manometry ☐ Ad hoc follow‐up by obstetrician ☐ Ad hoc follow‐up by colorectal surgeon ☐ Other
26. Please use the following space to add any other comments you may have relating to the primary repair of obstetric anal sphincter injuries
